# Continuous monitoring of aerial density and circadian rhythms of flying insects in a semi-urban environment

**DOI:** 10.1371/journal.pone.0260167

**Published:** 2021-11-18

**Authors:** Adrien P. Genoud, Gregory M. Williams, Benjamin P. Thomas

**Affiliations:** 1 Department of Physics, New Jersey Institute of Technology, Newark, New Jersey, United States of America; 2 Center for Vector Biology, Rutgers University, New Brunswick, New Jersey, United States of America; Uppsala University: Uppsala Universitet, SWEDEN

## Abstract

Although small in size, insects are a quintessential part of terrestrial ecosystems due to their large number and diversity. While captured insects can be thoroughly studied in laboratory conditions, their population dynamics and abundance in the wild remain largely unknown due to the lack of accurate methodologies to count them. Here, we present the results of a field experiment where the activity of insects has been monitored continuously over 3 months using an entomological stand-off optical sensor (ESOS). Because its near-infrared laser is imperceptible to insects, the instrument provides an unbiased and absolute measurement of the aerial density (flying insect/m^3^) with a temporal resolution down to the minute. Multiple clusters of insects are differentiated based on their wingbeat frequency and ratios between wing and body optical cross-sections. The collected data allowed for the study of the circadian rhythm and daily activities as well as the aerial density dynamic over the whole campaign for each cluster individually. These measurements have been compared with traps for validation of this new methodology. We believe that this new type of data can unlock many of the current limitations in the collection of entomological data, especially when studying the population dynamics of insects with large impacts on our society, such as pollinators or vectors of infectious diseases.

## Introduction

Insects, through their large diversity, numbers and biomass, play a crucial role in a variety of processes [1, 2]. Whether it is to study beneficial species, such as pollinators, or to implement mitigation methods for detrimental species, such as mosquitoes or locusts, fine scale measurements of insect’s behavior is critical. However, the monitoring of insect’s activity faces serious challenges, resulting in little quantitative data about their population dynamics. Monitoring change in insect distribution, diversity and abundance poses a formidable challenge to entomologists. Long-term estimates of population trends among insect species are difficult to implement, they require qualified personnel to collect and identify captured insects, often for years. The diversity, number, and size of insects are added difficulties making such endeavors labor and capital intensive. As a result, studies reporting observations of actual changes in population remain rare [3–8]. While immensely valuable, such studies are geographically limited and specific to just a few species of insects compared to the millions of discovered species. Furthermore, insects’ spatial distribution and abundance can rapidly vary with global climate change [9–12]. As a result, estimating trends in population of specific insect groups, both on a local or global scale, greatly suffers from our inability to collect entomological data.

Existing studies monitoring insect populations are often done using traps [13], which rely on counting the number of specimens captured to extrapolate on insect abundance. This methodology offers a highly accurate way to identify the specimen, which is key to study the diversity within an ecosystem. However, traps present some limitations for the evaluation of population dynamics. In most application, traps can only provide a relative estimate of the insect population [14] and generally of only the few species that are attracted by the bait in use. Indeed, the number of captured insects is a function of the collection efficiency and the effective range of the employed device, both of which being challenging to evaluate quantitatively and vary according to species, meteorological conditions and trap design [13, 15–18]. While a relative count can inform on population trends near a trap or a network of traps, the lack of an absolute metric related to the insect population precludes making any comparisons of the population observed by other measurements. Furthermore, the effective range of a trap may vary with time, attractants can potentially be more effective depending on wind conditions as well as competing stimuli in the vicinity of the trap, making population estimate not only relative but potentially biased as well. Finally, the identification process of each captured insect remains laborious. Surveillance campaigns are often limited by the number of insects they can identify, which in turn drastically limit the sample size. The time at which each insect is captured is often unknown or with poor temporal resolution, making traps ill-suited to monitor circadian rhythms, peak activities or behavioral patterns of insects in their natural habitat. We argue that the lack of entomological data discussed above is directly linked to the limitations of the current tools used to monitor insects in their natural habitat.

In this context, several research teams developed alternative approaches to tackle this issue. Among them, radar [19] and lidar [20] technologies have shown promising development over the years. Entomological radars have achieved significant results in the study of insects, in particular for high altitude migrations [21–23]. They benefit from covering large volume of air, making them capable of observing extremely large number of insects, close to two million in the study by Hu et al., 2016 [24]. However, large radar reflections from vegetation and ground features (clutters) prevent the radar methodology from operating at close proximity to the ground. Despite this limitation due to clutter, some studies of insect flight behavior at medium altitude have been achieved, as low as ~10 m [25], yet remain too high for the study of several key insect species. Alternative methods allow entomological radar to operate closer to the ground, in particular harmonic scanning radar [26], but it requires the use of a transponder attached to the studied specimen and as such, is of particular use for the study of flight behavior but limited for evaluating the number of specimens present in the wild. In parallel, infrared entomological lidars have gained significant traction [27–29]. Their wavelength, generally between 800 and 1500 nm, allows for the study of small insects as well as insect wing movements, enabling the retrieval of each insect’s wingbeat frequency. They can operate near ground level, where several key species dwell, and can cover a distance in the hundred-meter range [27–30].

In this contribution, we present measurements of the absolute aerial density of four clusters of flying insects for 80 consecutive days with a one-hour resolution using an entomological stand-off optical sensor (ESOS). This is, to the best of our knowledge, the first continuous recording of the absolute aerial density of flying insects for such an extended period. Because ESOS operates with a laser source emitting in the near infrared spectral range and does not use any attractant, the instrument is imperceptible to insects. Therefore, it limits the risk of biases that, as detailed earlier, traps can sometimes exhibit [13, 15–18]. In particular, the detection system of ESOS does not favor any specific insect families, species, age, or sex groups over another. The system operated continuously with minimal supervision, with a total down-time lower than 1.2% over the 80-day period and can operate in such manner for months. The system observed a total of 60,000 insect transits despite being in a semi-urban area with a relatively small number of insects (Secaucus, NJ, USA). Aerial densities of mosquitoes retrieved by the ESOS system were compared and were in agreement with the relative counts of several CDC Light traps (Centers for Disease Control and Prevention) in the area, which are one of the standard methods in the study of mosquito abundance. The continuous measurement of the aerial density from August 19^th^ to November 6^th^, 2020 allowed for the study of the aerial density dynamics, circadian rhythms and peak activities of each cluster of insects.

We believe that laser-based instruments combined with the presented methodology can unlock many of the current limitations in the collection of data in the study of insects and their behavior. This methodology has multiple features that are complementary to commonly used traps, such as the ability to 1) operate continuously for months 2) retrieve the absolute aerial density of multiple clusters of insects 3) have a temporal resolution potentially in the minute range 4) observe a number of insects at least an order of magnitude higher than traps, 5) do not present any bias in term of insect groups (family, species, sex, age) and 6) remains affordable and accessible to entomologists. Therefore, ESOS and traps could be used in conjunction where the specimens captured by the traps are analyzed and their characteristics identified precisely within the ESOS data.

The paper is organized as follow, first the methodology section presents the experiment and the instrument itself as well as the methodology and how raw data are processed. Then, the clusters and their respective aerial densities and circadian rhythms are displayed and discussed in the results section.

## Methodology

### Experiment and instruments

**Fig 1:**
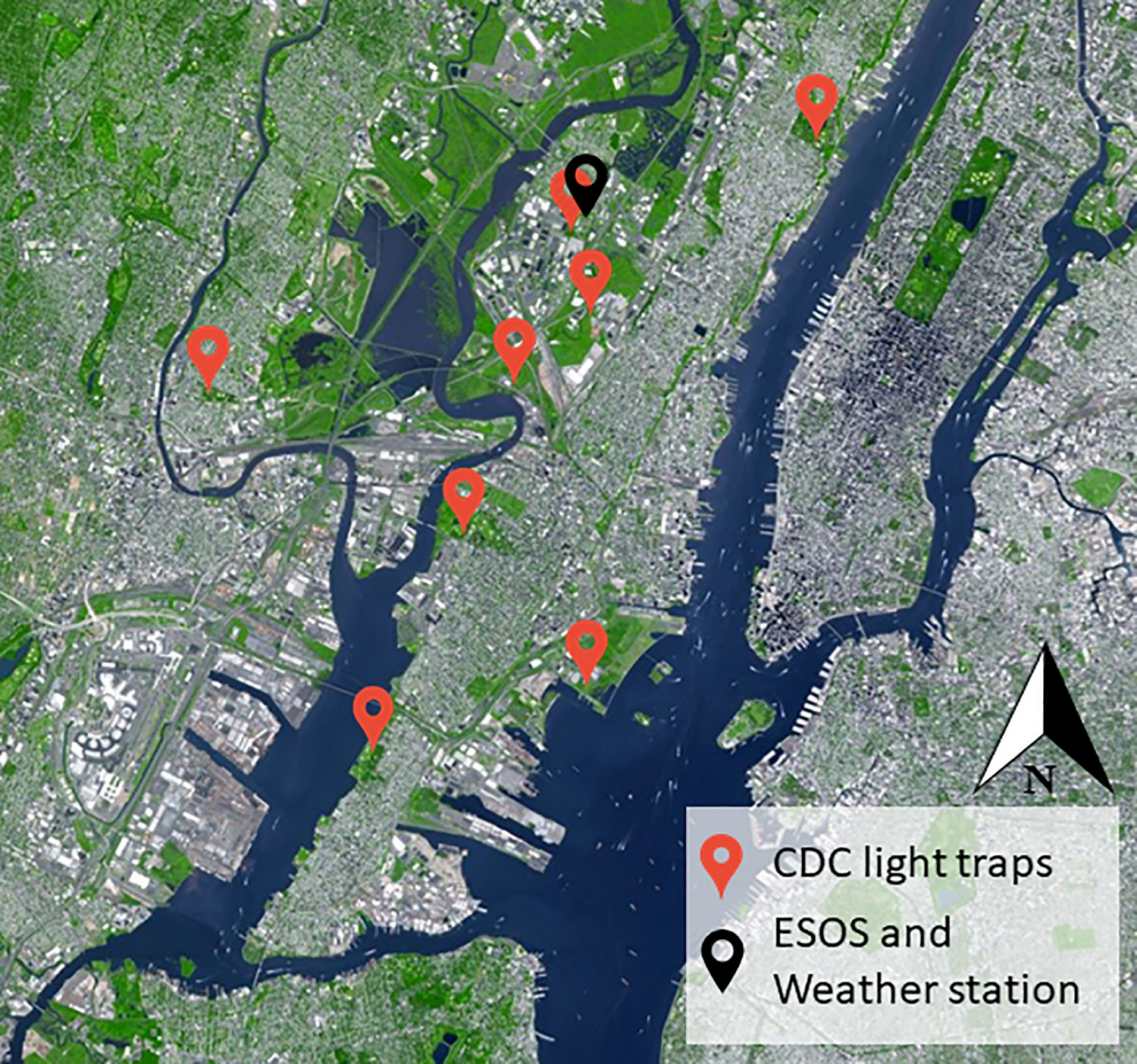
Aerial view of the experiment location (40°47’09.8"N 74°03’28.1"W) and its surroundings, the US Northeast megalopolis (Manhattan Island top right corner). The ESOS system and the co-located weather station are symbolized by a black marker. The position of the CDC light traps used for comparison are indicated by a red marker. Image courtesy of NASA’s Earth Observatory, NASA/GSFC/MITI/ERSDAC/JAROS, and U.S./Japan ASTER Science Team.

The ESOS instrument was deployed in a semi-urban environment, in a small patch of green within the city of Secaucus (Hudson County, NJ, USA) (Fig 1) in the US Northeast megalopolis. The field is approximately 40 x 10 m with tall grass bordered by a roughly 1 ha old-growth temperate woodlot. This specific location has been chosen for several reasons: 1) The field being part of the regular operation sites of one of the co-authors, no permit was required to access the field location, 2) The field is enclosed by a fence, reducing the risk of unwanted human interaction with the instrument, 3) The field allows for an easy access to the electrical grid while being in a patch of green.

**Fig 2:**
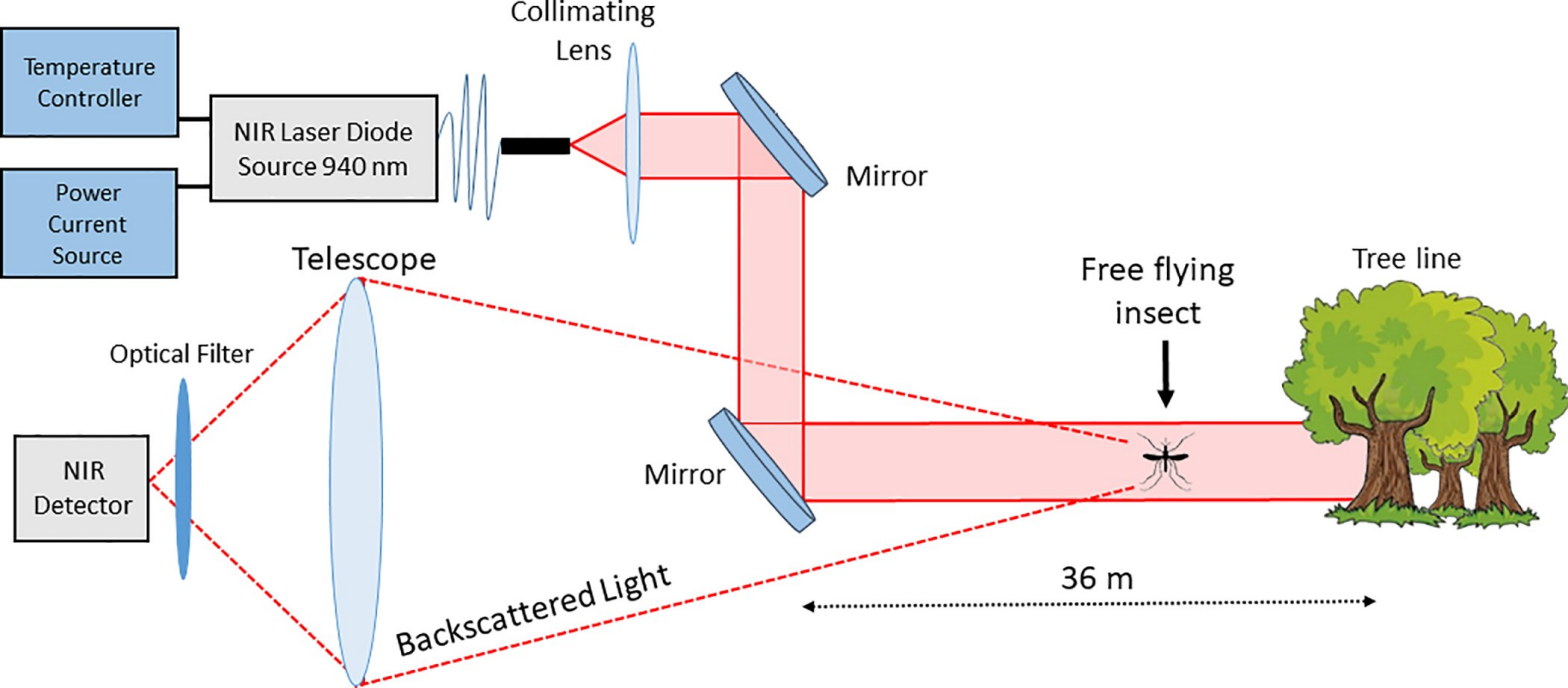
Optical layout of the entomological stand-off optical sensor (ESOS).

The ESOS system is a laser-based optical sensor that records the light backscattered by any targets that transit through its laser beam. Similarly to a lidar system, the laser beam and the telescope optical axis are coaxial and are both pointing in the same direction. As displayed in Fig 2, the telescope focuses the light backscattered by insects on an optical detector after passing through an optical filter to reduce the Sun light contribution. The laser source is a 4W continuous wave diode operating at 940 nm wavelength (L4-9891510-100M; Lumentum, Milpitas, CA) with an initial beam diameter of 2.54 cm and 3 mrad divergence. The telescope is a 25.4 cm converging lens and the detector an InGaAs amplified photodetector (InGaAs PDA20CS2; Thorlabs, Newton, NJ). The backscattered signal is recorded at a sampling frequency of 30,517 Hz by a 16-bit digitizer (M4i4420-x8; Spectrum, Stamford, Connecticut). The optical path is horizontal, starting about 25 cm above the ground near the emitter and finishing 36 m further in the forest approximately 2.5 m above the ground due to the slight inclination of the field. The instrument is powered by the electrical grid and uses less than 20W, plus approximately 80W for the computer used for the data acquisition. Finally, we estimate that a similar system can be reproduced for approximately $10,000, including the acquisition system.

The measurement campaign took place from August 19^th^ to November 6^th^, 2020. Over that period, the instrument was temporarily shut down every three to four days for checkups and routine verifications, resulting in an overall down time of 1.2%. At close proximity to the field, (Fig 1), a portable weather station (WS-1002-WIFI, Ambient Weather, Chandler, Arizona) was used to monitor several key environmental parameters, such as UV radiation, temperature, wind direction and speed, relative humidity, rainfall and atmospheric pressure.

In addition to the ESOS system, eight light traps (John W. Hock Co., Gainesville, FL) were operated across Hudson County, NJ, throughout the study period. The traps were deployed by the Hudson Regional Health Commission for monitoring mosquito populations. The traps used a 25-watt incandescent bulb to attract flying insects. A light sensor caused the trap to run every night from dusk to dawn. As insects flew near the bulb, a fan sucked them into a collection jar. The jar contained dichlorvos impregnated strips (Nuvan Prostrips, AMVAC, Los Angeles, CA) to kill the insects. Specimens were collected two times a week and returned to the laboratory for identification. All Culicidae were retained and identified to the species level while other insect families were discarded.

### Data analysis

**Fig 3:**
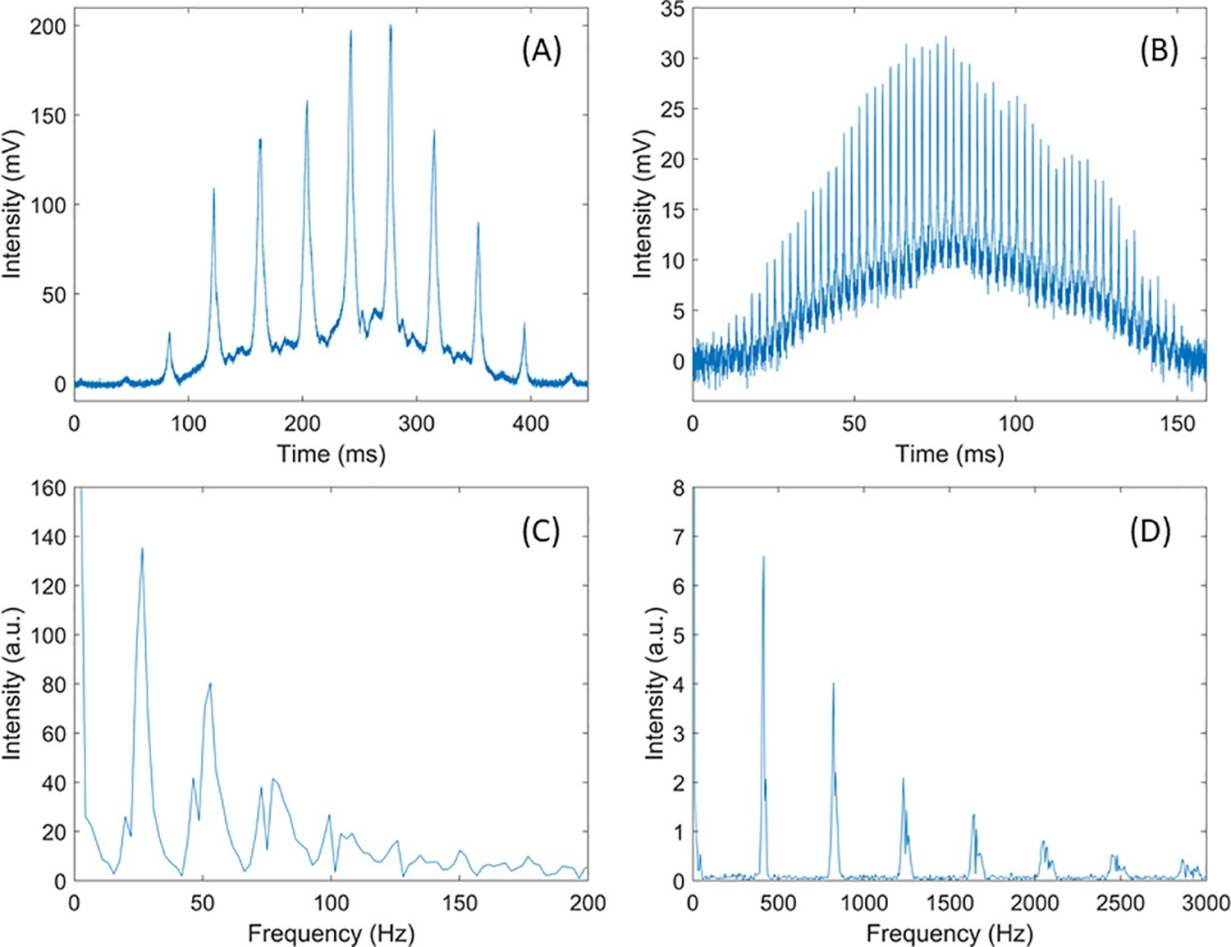
Examples of insect signals as they transit through the laser beam of the ESOS system (A and B) along with their frequency analysis through fast Fourier transform (C and D).

Fig 3A and 3B show two examples of backscattered signals from insects transiting through the laser beam. Both signals display an overall gaussian shape due to the spatial profile of the laser beam. In addition, sharp intensity peaks in the backscattered signals are visible due to the rapid movement of insects’ wings causing the optical cross section of the insect to change. These oscillations provide information on the wingbeat frequency of the specimen. As shown in Fig 3C and 3D, a frequency analysis of the signal, using a fast Fourier transform, inform on the wingbeat frequency (the fundamental) and its harmonics. Fig 3A and 3C show a wingbeat frequency of 26.5 Hz, typical of large insects such as moths, while Fig 3B and 3D show a much higher wingbeat frequency of 410 Hz, typical of female mosquitoes [31, 32].

The frequency components of the backscattered signals allow for the discrimination between insects and falling leaves or drifting pollen grains. Contrary to other targets, an insect’s event will display a periodic oscillation due to the apparent cross section of the wings changing over time. The determination of the wingbeat frequency is achieved by 1) scanning the frequency spectrum for any peak exceeding a certain intensity threshold, 2) identifying if a series of peaks creates a harmonic series, and 3) if the frequency range between harmonics corresponds to one of the peak’s frequency, this frequency is selected as the fundamental frequency, i.e., the wingbeat frequency of the insect.

In addition to retrieving the wingbeat frequency, backscattered insect’s signals, such as the ones presented in Fig 3, can be used to evaluate the ratio between the backscattering cross section of the wings versus the backscattering cross section of the body, here after referred to as *wing to body ratio*. The wing and body contributions can be separated by interpolation of local minimums of the signal [31] or by parametrization [33]. Because the distance between instrument and insect is unknown, the system cannot retrieve the absolute cross section. However, the ratio of the body and wing cross section can be retrieved and is expected to vary from one insect species to the other based on the differences in morphological characteristics and wing/body backscattering efficiency. Typically, a signal from an insect such as a butterfly will have a large wing to body ratio, potentially between 5 and 20, while this same ratio for mosquitoes is typically below 1.5.

Finally, the transit time (Δt) can be evaluated from the signal and is defined as the time it takes for the insect to fly through the laser beam. It can vary from a few milliseconds for insects crossing the beam around its edges up to a second for insects flying along the optical axis. As detailed in the following section, this transit time information can be used to evaluate the aerial density of insects that are flying in the area.

### Aerial density

Entomological photonic sensors do not directly inform on the actual aerial density or population size, and often the number of transits is used as a relative metric to study insect’s population. However, we argue that the number of transits, or transit counts, is not a proper metric to evaluate insect population. First, it is function of the instrument itself, i.e. a longer range or larger laser beam would result in an increased number of transits, making the comparison of data collected by different instruments or different campaigns less relevant. Secondly, fast and highly mobile insects might contribute a disproportionately high number of transit counts since they are more likely to interact with the instrument. On the other hand, slower paced insects might account for fewer transits despite potentially occurring in greater numbers. For similar reasons, high winds may artificially modify the velocity of flying insects and possibly change the number of transits, adding a systematic error to transit counts.

This campaign takes advantage of a methodology described in length in A. Genoud et al, 2020 [34], that allows for the retrieval of the aerial density of insects as a function of time from the data collected by photonic sensors. We believe that this is a crucial step in the analysis as it allows for the retrieval of an absolute and unbiased metric expressed as the number of flying insects per cubic meter, which is directly related to the actual insect population. Briefly, the methodology relies on the sum of the transit time instead of the number of transits which is then normalized by the time of observation and the probed volume of air. The aerial density of flying insects *ρ*, expressed in m^-3^ is evaluated using Eq 1:

$$\rho =\frac{\sum_{i}{\Delta t}_{i}}{T\cdot \Delta V}$$ (1)

Where ΔV is the volume probed by the laser beam in m^3^, and T is the duration of observation during which the aerial density is to be determined. Finally, ∑*_i_Δt_i_* is the sum of all transit times *Δt_i_* of all insects (or cluster of insects, see Results section: Clustering) during the duration T. The aerial density *ρ* can be evaluated as a function of time where T becomes the temporal resolution associated to the aerial density.

## Results

Over 80 days in this semi-urban environment, the instrument detected a total of 72,975 transit signals, from which 59,105 (80.1%) presented acceptable harmonic series in the frequency domains and were retained as originating from insects for further analysis. From each of these transits, the wingbeat frequency, wing to body ratio and transit time were recorded together with the data collected by the weather station.

### Correction in temperature

**Fig 4:**
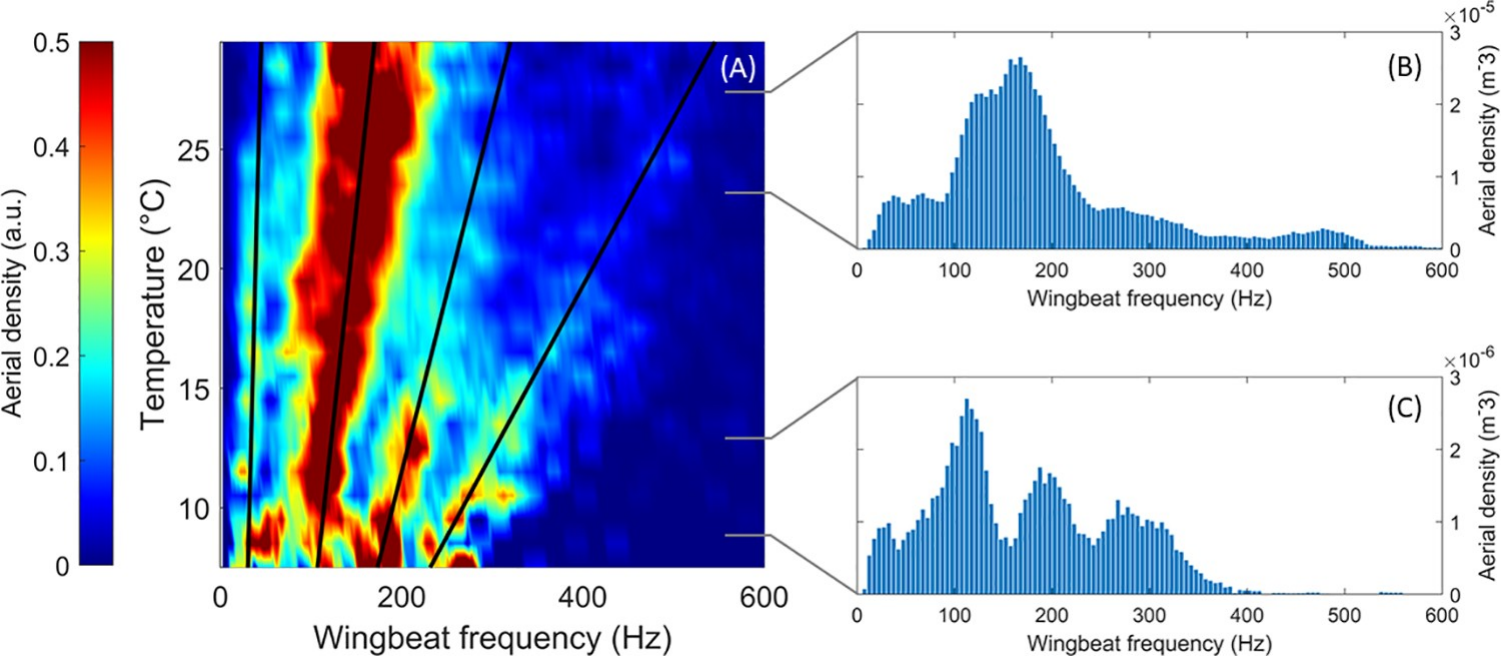
Relative aerial density per bin of temperature and wingbeat frequency (A) along with the wingbeat frequency distribution for two ranges of temperature, 22 to 26°C and 8 to 12°C (respectively B and C). On figure A the aerial density was rescaled from 0 to 1, for each temperature bin by normalizing with the maximal value in order to emphasize local maximums. Black lines represent the linear fit of local maximal for each of the four insects cluster, illustrating the drift of the wingbeat frequency with the temperature.

The wingbeat frequency of many insect species is known to change with air temperature due to the change in air density as well as the change in body temperature. While the relationship between ambient temperature and wingbeat frequency is complex and beyond the scope of this paper, it appears to be impacted by the size of the insects as well as the wingbeat frequency itself [35, 36]. With the field experiment starting in August and finishing in November, the air temperature observed during the experiment, measured from a local weather station, went from a minimum of -2.2°C to a maximum of 35.9°C. A local measurement of the air temperature allows for the correction of wingbeat frequency measurements to avoid any source of bias due to temperature. Fig 4A presents a color plot of the measured wingbeat frequency as a function of the ambient temperature, while Fig 4B and 4C are the wingbeat frequency distributions for two ranges of temperature, 22 to 26°C and 8 to 12°C respectively. Fig 4A shows that the correction in wingbeat frequency increases with the temperature as well as the wingbeat frequency itself, i.e. the greater the wingbeat frequency, the greater the correction terms. This is also illustrated by the change of the slope with f_0_ in Eq 2.

**Fig 5:**
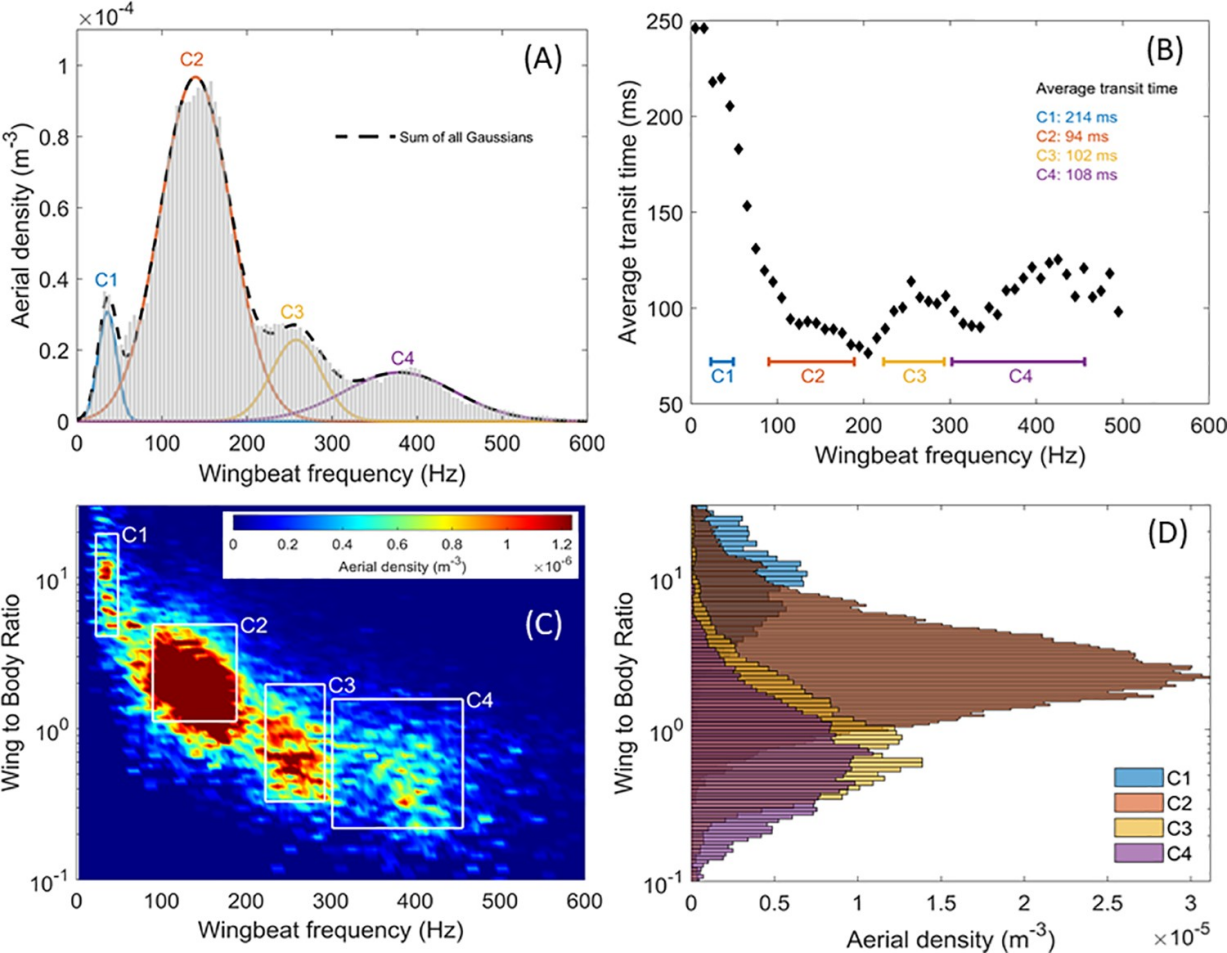
Distribution of the wingbeat frequency corrected in temperature (A) with four Gaussian fits corresponding to each insect cluster, and the total reconstructed gaussian fit (dashed black line). Average transit time in function of the wingbeat frequency (B), the different cluster range are displayed, and their mean transit time indicated. Aerial density per bin of wing to body ratio and wingbeat frequency (C), the boundaries of the four insect clusters C1 to C4 are indicted by white lines. Distribution of the wing to body ratio separated per cluster (D).

From Fig 4B and 4C, one can observe both the broadening of the clusters and the increase of the wingbeat frequency with the temperature. At every recorded temperature, the wingbeat frequency distribution presents four clusters (See Results section: Clustering). The black lines in Fig 4A show the linear fit of the local maximum for each of the four clusters at different temperatures. A correction in temperature applied to the measured wingbeat frequency is derived from these linear fits and the resulting distribution of all wingbeat frequencies corrected in temperature is displayed in Fig 5A. The empirical correction is presented in Eq 2 and was experimentally derived from a power law fit on the slope coefficients of the aforementioned linear fits of the maximums.


$$f_{corr}=f_{0}-(T_{0}-T_{ref})\cdot (0.001189\cdot f_{0}^{1.561})$$ (2)

Where f_corr_ is the wingbeat frequency corrected in temperature, f_0_ the original wingbeat frequency retrieved from the fast Fourier transform harmonic analysis, T_0_ the temperature at the time of measurement and T_ref_ the reference temperature, chosen to be 20°C. This correction term is in good agreement with the finding of other studies on the relationship between wingbeat frequency and ambient air temperature. In the study by Unwin et al, 1984 [36], a variation of the wingbeat frequency between 1.2 to 4.6% per degree was found for the stingless bee *Trigona jaty* (Hymenoptera: Apidae). Using Eq 2, our correction for insects with the same fundamental frequency as *T*. *jaty* would be 2.1% per degree. Similarly, in the study conducted by Oertli, 1989 [37], the correction for the click beetle *Agriotes sputator* (Coleoptera: Elateridae) and the eastern firefly *Photinus pyralis* (Coleoptera: Lampyridae) was found to be between 0.1 to 2.2% and 0.9 to 1.6% per degree, respectively, while Eq 2 yields a correction of 1.6 and 1.2%, respectively.

### Clustering

Despite improvements in the last few years [32, 38–40], species level identification from infrared optical sensors has yet to reach practical reliability for field campaigns. However, it is possible to identify different clusters of insects with shared characteristics, such as wingbeat frequency, wing to body ratio and activity patterns. Clusters still provide relevant information, especially for mosquito species since their high wingbeat frequency makes their identification from other insect Families more reliable. Fig 5 displays the four distinct insect clusters C1, C2, C3 and C4 that have been identified.

Fig 5A shows that the wingbeat frequency distribution of all events can be effectively fitted by four Gaussian distributions, which suggest the presence of a least four insect clusters. The boundaries of each cluster in terms of wingbeat frequency are defined by the full width at half maximum of each Gaussian fit. Fig 5D displays the wing to body ratio of each cluster, showing a normal distribution. The boundaries of each cluster in terms of wing to body ratio are defined on the same model as the ones for the wingbeat frequency, i.e., full width at half maximum. It is interesting to note that the wing to body ratio distributions of each cluster do overlap, especially for cluster C3 and C4. This suggests morphological similarities between the insects present in both clusters. Fig 5C shows the aerial density as a function of the wing to body ratio and wingbeat frequency. The four clusters and their boundaries are illustrated by the white boxes and labeled C1 to C4. Finally, the average transit time as a function of the wingbeat frequency is presented in Fig 5B. Because insects may change directions multiple times while within the beam or fly along the optical axis, the path length is unknown and, thus, the flight velocity is unknown. However, once averaged, transit times as a function of wingbeat frequency indicate that some clusters include insects with different flight speed, with C1 having the highest transit time, suggesting slow insects and C2 with the lowest transit time, suggesting faster insects.

This analysis suggests the presence of at least four insect clusters that possess sufficiently distinct characteristics to be discriminated from one another. Within some of these clusters, it is likely for more than a single insect family to be present and a more refined analysis or experimental improvement are needed to discriminate down to a family or species level. While it is beyond the current capability of this instrument to identify with high certainty the family and species present in each cluster, the information available can be coupled with the information collected from insects captured in traps in the area. Based on the measured wingbeat frequency and the wing to body ratio, the following paragraphs offer suggestions as to which families/species could be found in each cluster:

- Cluster C1: The first cluster is composed of insects with low wingbeat frequency, between 23 and 49 Hz, large wing to body optical cross section ratio between 4.1 and 19.5 and low velocity with an average transit time of 214 ms which is almost twice as every other clusters. This cluster may include crane flies (Diptera: Tipluidae), lacewings (Neuroptera: Chrysopidae), as well as moths and butterflies (Lepidoptera).
- Cluster C2: The second cluster is composed of insects with wingbeat frequency, between 90 and 189 Hz, medium wing to body ratio between 1.1 and 4.9 and the highest velocity with an average transit time of 94 ms, which is the lowest among all clusters. This cluster may include dragonflies and damselflies (Odonata), wasps (Hymenoptera: Vespidae), beetles (Coleoptera) and flies (Diptera).
- Cluster C3: The third cluster is composed of insects with wingbeat frequency, between 223 and 293 Hz, small wing to body ratio between 0.33 and 2.0 and an average transit time of 102 ms. This cluster may include bees (Hymenoptera: Apoidea), leafhoppers (Homoptera: Cicadellidae) and flies (Diptera)
- Cluster C4: The fourth cluster is composed of insects with the highest wingbeat frequency, between 302 and 456 Hz, the smallest wing to body ratio between 0.15 and 1.3 and an average transit time of 108 ms. This cluster may include mosquitoes (Diptera: Culicidae) and midges (Diptera: Chironomidae).

### Circadian rhythm

**Fig 6:**
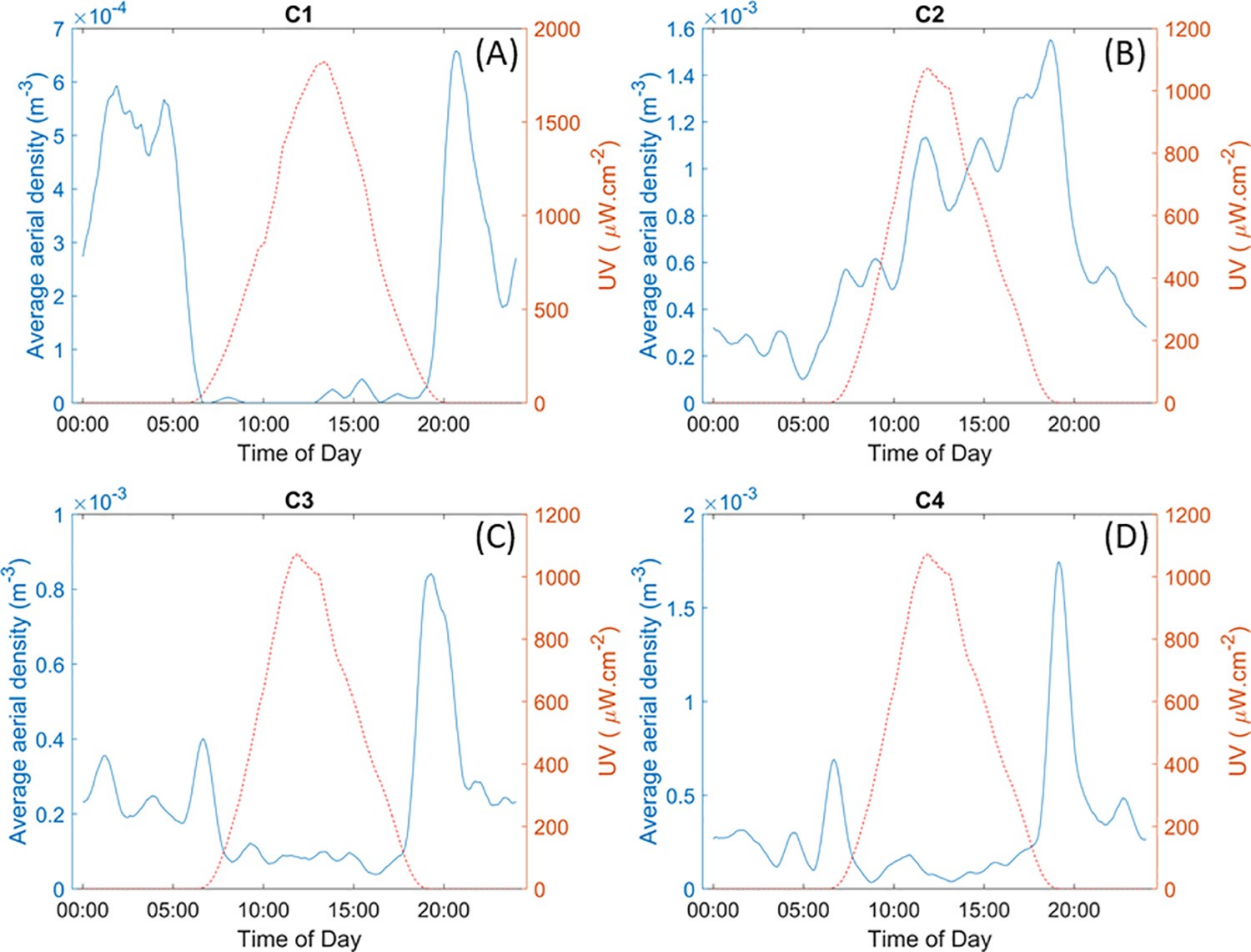
Typical circadian rhythm for cluster C1, C2, C3 and C4 (respectively A, B, C and D). The dotted line displays the UV radiation measured on the field which relates to the sun activity. The aerial density is the one hour sliding average over 14 consecutive days of measurements.

The ESOS system benefits from a high temporal resolution when compared to traps, possibly down to a single minute resolution. As discussed in Genoud et al, 2020 [34], the chosen temporal resolution directly impacts the uncertainty of the retrieved aerial density. As it relies on a statistical analysis, see Eq 1, the estimated aerial density is subject to stochastic fluctuations. These fluctuations, or uncertainties, are inversely proportional to the number of transit signals [34]. Therefore, a smaller time resolution implies a higher uncertainty. Similarly, fewer flying insects and a smaller probed volume results in greater uncertainties on the aerial density. This entails that, for a one-minute resolution to be meaningful, the probed volume and/or the aerial density of insects must be sufficiently large. In this study, of a semi-urban environment with only 36 m of unobstructed range, the probed volume and insect abundance did not allow for such a resolution to be used with sufficient accuracy. Therefore, the one-minute resolution was averaged over a sliding window of one hour to mitigate the uncertainty by increasing the parameter T of Eq 1. Fig 6 shows the circadian rhythm of each of the four clusters, in the form of the aerial density over 24h, obtained from the application of a one hour sliding window over 2 weeks of continuous recording.

**Fig 7:**
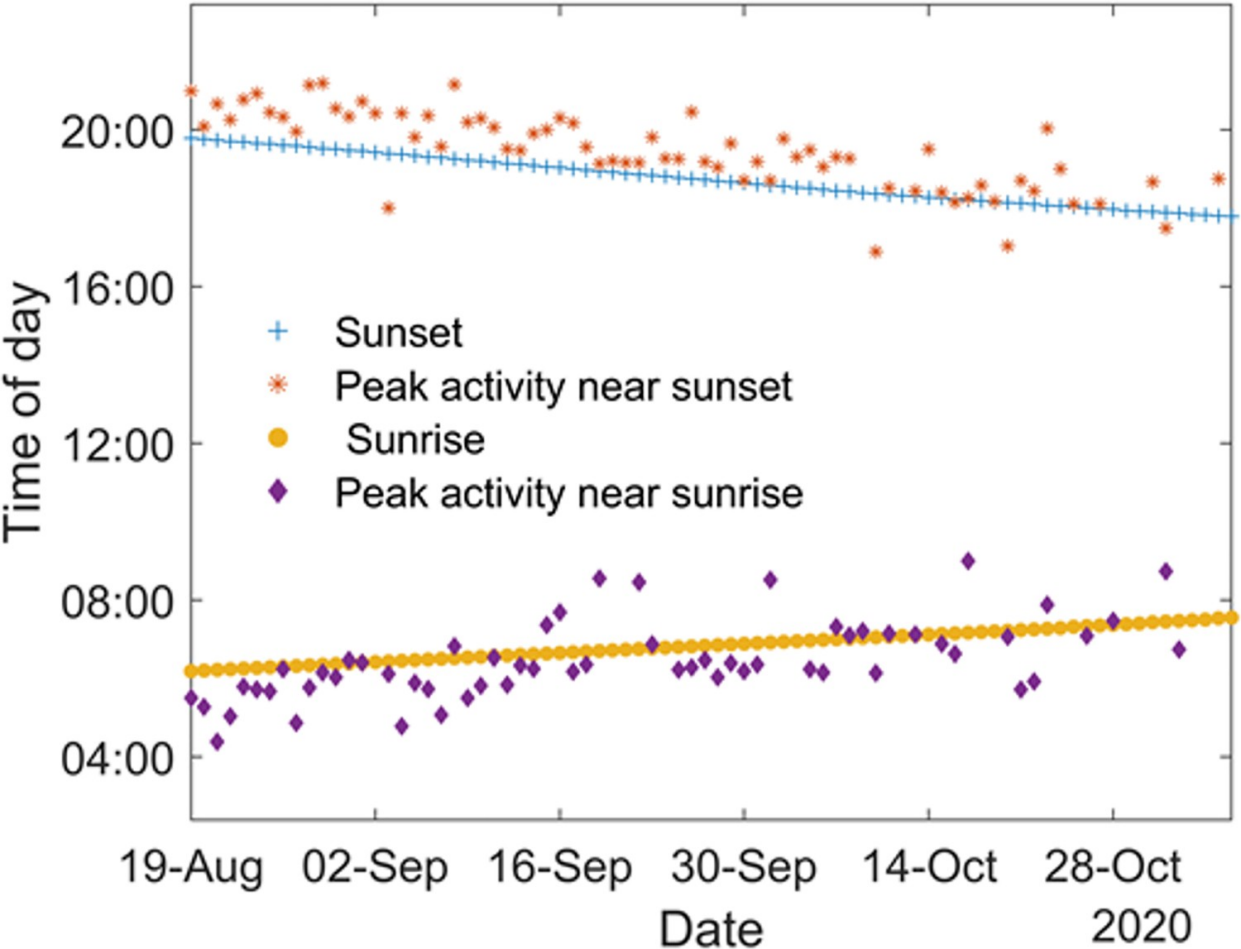
Peaks of mosquito activity (Cluster C4) near sunset (orange stars) and sunrise (purple diamond) in relation with the actual sunset (blue plus) and sunrise (yellow disk) time.

The circadian rhythm of the second cluster, Fig 6B, displays activity predominantly during the day, which provides additional information on cluster composition, as it must include mainly diurnal insects. Clusters C1 and C3 show activity mainly during the night with peaks of activity at sunset and sundown. Finally, cluster C4 displays activity almost entirely limited to dusk and dawn, which is consistent with the cluster being mainly composed of mosquito species. The peaks of activity of the cluster C4, henceforth referred to as the mosquito cluster, near sunset and sunrise are furthermore studied and the results presented in Fig 7. As shown in Fig 7, the peak of activity of this cluster follows the shift in time of sunset and sunrise throughout the season, showing that the behavior of the mosquito species present on the field is correlated with the sun activity as expected. On average, the peak of activity of mosquitoes near sunset is 38 minutes after the actual sunset time, as provided by the NOAA Solar calculator, while the peak of activity near sunrise is on average 17 minutes before the actual sunrise. However, using those circadian rhythms must be done carefully. Without a species level identification, each cluster is very likely to encompass several distinct species and as such can only display the overall circadian rhythm of the entire cluster. This amalgamated information could hide the specific behavior of the individual species present within the cluster.

### Aerial densities over 80 days

**Fig 8:**
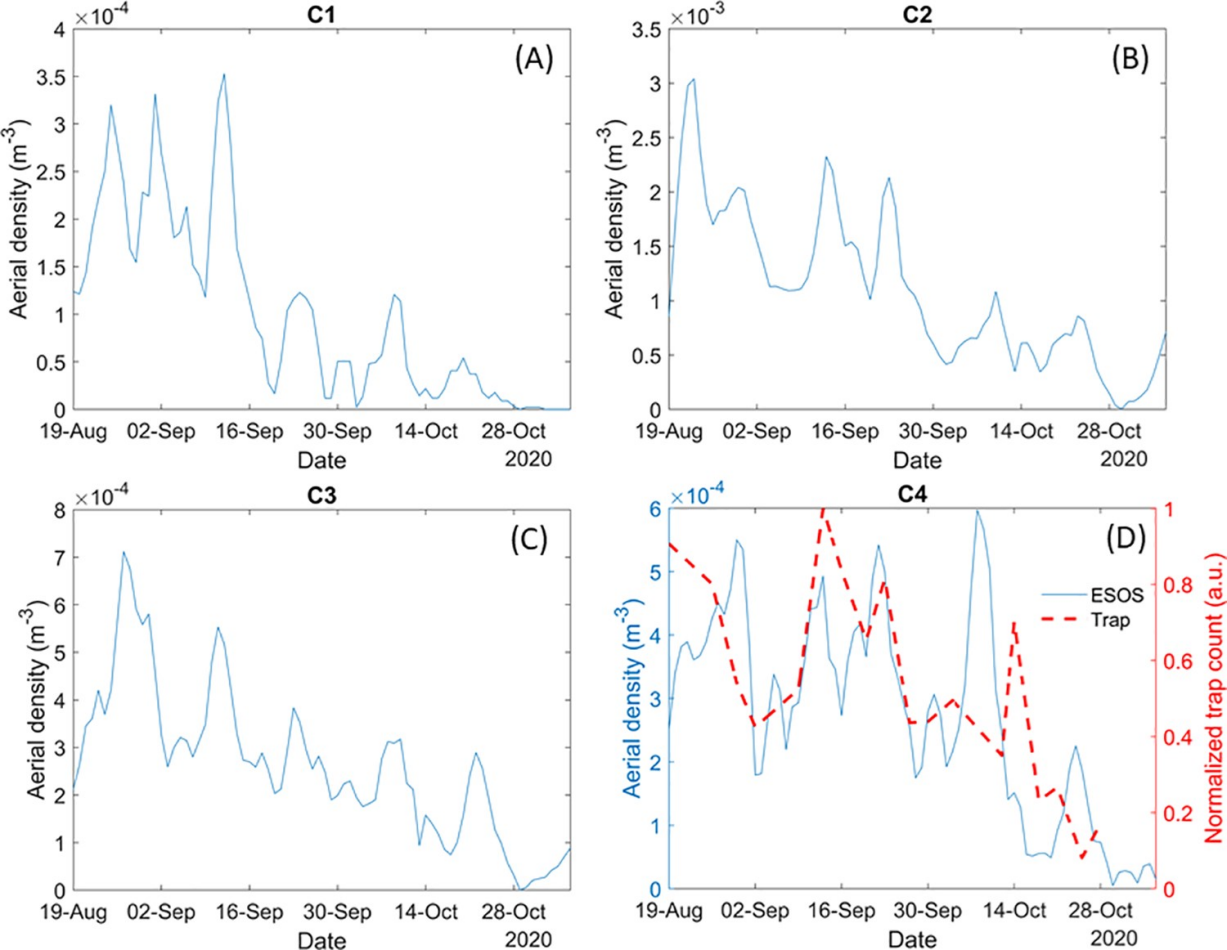
Evolution of the aerial density for clusters C1, C2, C3 and C4 (respectively A, B, C and D) over the 80 days of the measurement campaign. Figure D also displays the normalized trap count (red dashed) of nearby traps for comparison purposes.

The aerial density for each day has been measured by averaging the aerial density over 24h for every cluster and is presented in Fig 8.

There is a clear overall decrease in insect aerial density over time for all clusters. This is likely due to the temperature decrease and day length reduction as winter approaches. In addition, we observe cycles of increase and decrease of aerial density throughout the measurement campaign. The reasons for those cycles may be explained by the natural cyclical variation of population and environmental factors such as humidity and rainfall. These cycles may also correspond to the emergence and disappearance of different short lived and highly seasonal species. The second cluster is by far the most abundant, in terms of aerial density, with a density five to ten time greater than any other cluster. It is the cluster that most likely encompassed the greater number of distinct species, as almost all of the predominantly diurnal species are within this cluster, although its exact composition cannot be entirely defined by the results of this study. The remaining three clusters all have comparable aerial density. However, cluster four only encompasses mosquito and, to a lesser extent midge species, with their typically high wingbeat frequency, while cluster one and three are likely to be much more diverse, in term of species.

As the ESOS methodology is new, it calls for a validation by comparison with well tested and widely approved methodologies. The study of insects is often conducted with traps, which counts the number of captured specimens to study the population of insects. Every few days traps (described in the methodology section) were emptied and the number of mosquitoes captured were counted by the Hudson Regional Health Commission. This gives access to the number of specimens retrieved from traps on different days. In Fig 8D, the number of mosquitoes captured at each trap location is normalized and the average is then displayed (red dashed line) with the aerial density of the mosquito cluster C4 retrieved by the ESOS system. While the captured mosquitoes have been identified at the species level, ESOS cannot discriminate at the species level, thus Fig 8D presents the trap count for all species of mosquitoes combined so it can be compared to the ESOS data.

This figure shows a good agreement between both methodologies, with a similar overall downward trend and cyclical variation between the two methods. This demonstrates that the aerial density retrieved by the ESOS system is coherent with the count of traps, which is currently one of the standard methods for monitoring mosquito populations.

## Conclusion

This study presents the results of a 3-month long campaign where the aerial density of flying insects has been monitored using an entomological stand-off optical sensor (ESOS). The system operates in the near-infrared spectral range and observed near 60,000 events of flying insects transiting through its field of view. The system identified four clusters of insects from their wingbeat frequency as well as a ratio of their wing to body optical cross section. The methodology presented in this contribution allowed for the retrieval of the absolute aerial density expressed in number of flying insects per m^3^ of air with a temporal resolution in the minute range, showing the decline of insect population as winter arrives. In addition, the data collected was used to study the circadian rhythm of each cluster individually and detect daily peak activities which, in the case of mosquitoes, is changing together with sunset and sunrise times. With the difficulty to monitor insect population, we believe that this methodology combined with the high accuracy for identification provided by traps could significantly increase the data available in the field of entomology, especially to study how insect populations are reacting to various stimuli/events such as climate change, pesticide applications and other mitigations technics of pests, to name a few. The use of optical sensors remains fairly new in the field of entomology, with potentially room for further improvements regarding the identification capability of each specimen transiting through their laser beam. Moreover, an increase of the volume probed by such instruments could even further reduce the temporal resolution at which the aerial density can be studied, potentially unlocking a greater understanding of short time insect behavior and dynamics. As such, the increase of the probed volume is to be prioritized for the next generation of ESOS systems. This improvement would reduce the uncertainty of the measurements, unlocking greater temporal resolution, while increasing the number of events observed. In addition, the fact that this methodology can retrieve an absolute measurement of the aerial density allows for comparisons with future campaigns, years after years and between different locations, opening the path for new types of long-term studies of insect population. In view of the many species driven to extinction in modern age, this type of methodology could be a powerful asset to monitor and protect this large and diverse fauna.
